# Intraoperative Scoring System to Assess the Difficult Laparoscopic Cholecystectomy: A Prospective Study From a Tertiary Care Centre

**DOI:** 10.7759/cureus.35767

**Published:** 2023-03-04

**Authors:** Aishwarya Pal, Prabhjot S Ahluwalia, Kanika Sachdeva, Richam Kashyap

**Affiliations:** 1 General Surgery, Pandit Bhagwat Dayal Sharma Post Graduate Institute of Medical Sciences, Rohtak, IND; 2 General Surgery, Manipal Hospitals, Delhi, IND; 3 General Surgery, SGT Medical College Hospital & Research Institute, Gurgaon, IND

**Keywords:** preoperative scoring system, intraoperative scoring system, common bile duct, gallbladder, laparoscopic cholecystectomy

## Abstract

Background

In comparison to a traditional cholecystectomy (open), the laparoscopic cholecystectomy approach provides a number of benefits and has been demonstrated in some studies to have a greater complication rate. The conversion rate from laparoscopic to open surgery ranged between 2% and 15%. A preoperative scoring or grading system (based on age, sex, history, clinical examination, laboratory, and sonographic results) was developed by Nassar et al., to anticipate the challenge of laparoscopic cholecystectomy. So, we conducted this study to assess the degree of difficulty in faced during laparoscopic cholecystectomy using an intraoperative scoring system and validate the same using the preoperative scoring system.

Methods

We conducted this study in the department of General Surgery among 105 patients who underwent laparoscopic cholecystectomy during the defined study period of one year. We performed the preoperative workup for all patients. A preoperative scoring or grading system developed by Nassar et al., in 2020 was used. In our study, laparoscopic cholecystectomy was performed by surgeons having a minimum of eight years of hands-on experience in laparoscopic surgeries. An intraoperative scoring or grading system for the degree of difficulty during laparoscopic cholecystectomy, developed by Sugrue et al., in 2015 was used. The Chi-square test was applied to assess the association between preoperative variables and the intraoperative score grading. We have also performed the receiver operating characteristic (ROC) curve analysis to validate the preoperative score in predicting the intraoperative findings. All tests were considered statically significant if the p-value was < 0.05.

Results

In our study, a total of 105 patients were included in the study and the mean age of patients was 57.6±16.4 years. The male patients were 58.1% and the remaining 41.9% were females. The primary diagnosis was cholecystitis among 44.8% of patients and 2.9% of patients were diagnosed with pancreatitis. Among enrolled patients, laparoscopic cholecystectomy was done on an emergency basis among 2.9% of subjects. During the laparoscopic cholecystectomy, among 21.0% and 30.5% of patients, there was a severe and extreme degree of difficulty respectively. In our study, the conversion rate from laparoscopic to open cholecystectomy was 8.6%. In our study, we found that at a preoperative score of 6, the sensitivity and specificity for predicting easy cases were 88.2% and 73.8%, respectively, and had an accuracy of 88.6% for easy cases and 68.5% for difficult cases.

Conclusion

When grading the difficulties of doing a laparoscopic cholecystectomy and determining the severity of cholecystitis, this intraoperative scoring system is effective and accurate. Additionally, it signifies the need for conversion from laparoscopic to open cholecystectomy in cases of severe cholecystitis.

## Introduction

The most common surgical intervention carried out in the biliary tract is a cholecystectomy, which involves removing the gallbladder (GB) due to inflammation or stone [1]. The preferred technique and established gold standard for the treatment of symptomatic cholelithiasis (gallstones) is laparoscopic cholecystectomy [1,2]. In comparison to a traditional cholecystectomy (open), this surgical approach provides a number of benefits, including lesser trauma or pain, reduced duration of hospital stays, a superior aesthetic (cosmetic) result, and faster healing [3,4].

Laparoscopic cholecystectomy, in contrast to traditional cholecystectomy (open), has been demonstrated in some studies to have a greater complication rate. Common bile duct (CBD) injury, leakage of bile, perforation of the GB, and damage to the structures (vascular/visceral) while using a Veress needle/trocar, are the common complications. There are as well as additional complications like foreign inclusions (foreign body), adhesions, collection (perihepatic), blood clot, metastatic deposits (port-site), the coughing up of gallstones (cholelithoptysis), fistula (external biliary), and sepsis of wound [1,3].

The conversion rate from laparoscopic to open surgery ranged between 2% and 15% in the initial days of the method. The conversion rate decreased to about 1% to 6% after years of studying and mastering the laparoscopic method and gaining experience with surgeons. Due to different difficulties posed throughout the procedure, this conversion was an effort to minimize complications [5]. When there are dense adhesions in Calot's triangle, a fistula (cholecystoduodenal/cholecystogastric), surgical history of the abdomen (upper) or cholecystostomy, a Mirizzi's syndrome, and empyematous/inflamed/gangrenous GB, the difficulty is taken into account [1,6].

An intraoperative scoring or grading system for the degree of difficulty during laparoscopic cholecystectomy, developed by Sugrue et al., in 2015 is based on intraoperative findings such as the GB appearance including contraction/distention of GB and degree of adhesions; access to the peritoneal cavity; Calot’s triangle dissection time period; and any complications (septic/local) based on scores, the patients are graded into four categories i.e., Mild (<2), Moderate (2-4), Severe (5-7), and Extreme (8-10) [7]. A preoperative scoring system was developed by Nassar et al. (based on age, sex, history, clinical examination, laboratory, and sonographic results), and it is then compared with the intraoperative score to anticipate the difficulty of laparoscopic cholecystectomy [8,9]. This enables surgeons to select a surgical technique that minimizes complications, conversion rate to open cholecystectomy, or medical expenses and to counsel patients on the same. So, we conducted this study to assess the degree of difficulty faced during laparoscopic cholecystectomy using an intraoperative scoring system and validate the same using a preoperative scoring system.

## Materials and methods

We conducted our study for a period of one year (June 2021 to May 2022) in the department of General Surgery of a government tertiary care hospital in North India after obtaining ethical approval from the institutional ethical committee (IEC). The study participants in our study were the patients who had undergone laparoscopic cholecystectomy during the defined study period and they were enrolled in the study after obtaining written informed consent either from patients or attendees (family/friends/relatives). As our study was prospective in nature, the patients were enrolled in our study consecutively over a period of one year and over this one-year period, 105 patients were enrolled in the study.

We performed the preoperative workup for all patients. A preoperative scoring or grading system (based on age, sex, history, clinical examination, laboratory, and sonographic results), developed by Nassar et al., in 2020 [10] was used, and based on scores the patients were graded into three categories i.e., Low risk (<2); Intermediate risk (2-6), and High risk (7-19) (Table 1). In our study, the laparoscopic cholecystectomy was performed by surgeons (all units) having a minimum of eight years of hands-on experience in laparoscopic surgeries. The surgeons created a Carbon dioxide (CO_2_) pneumoperitoneum of 10mmHg pressure using a Veress needle inserted from infraumbilical site/palmer’s point and surgery was done using two each 5mm and 10mm ports.

**Table 1:** Preoperative grading system for difficult laparoscopic cholecystectomy ASA: American Society of Anesthesiologists; ERCP: endoscopic retrograde cholangiopancreatography

Age (years)	
<40	0
40 and above	1
Gender	
Female	0
Male	1
ASA classification	
1	0
2	1
3	2
4	7
Primary diagnosis	
Pancreatitis	0
Biliary colic	0
Choledocholithiasis	1
Cholecystitis	4
Thick-walled gallbladder (3mm or more)	
No	0
Yes	2
Common biliary duct dilation (>6mm)	
No	0
Yes	1
Pre-operative ERCP	
No	0
Yes	1
Type of admission	
Elective	0
Delayed	1
Emergency	2
Degree of difficulty	
Low risk	0-1
Intermediate risk	2-6
High risk	7-19

During the intraoperative period, the surgeons observed and noted the GB appearance including contraction/distention of GB and degree of adhesions; access to the peritoneal cavity; Calot’s triangle dissection time period; and any complications (septic/local). An intraoperative scoring or grading system for the degree of difficulty during laparoscopic cholecystectomy, developed by Sugrue et al., in 2015 [7] was used, and based on scores the patients were graded into four categories i.e., Mild (<2), Moderate (2-4), Severe (5-7), and Extreme (8-10) (Table 2).

**Table 2:** Intraoperative grading system for difficult laparoscopic cholecystectomy BMI: body mass index

Gallbladder appearance	
No adhesions	0
Adhesions	1
Adhesions < 50% and completely buried Gallbladder	2
Gallbladder is completely buried in adhesion	3
Distension/Contraction	
Distended Gallbladder (or contracted shriveled G Gallbladder)	1
Unable to grasp with atraumatic laparoscopic forceps	1
Stone ≥1 cm impacted in Hartman’s Pouch	1
Access	
BMI >30	1
Adhesions from previous surgery limiting access	1
Severe Sepsis/Complications	
Bile or Pus outside Gallbladder	1
Time to identify cystic artery and duct >90 minutes	1
Degree of difficulty	
Mild	<2
Moderate	2-4
Severe	5-7
Extreme	8-10

Statistical analysis

We have entered the data obtained in the performed proforma into the MS excel sheet. The data was coded appropriately for the analysis in the Statistical Product and Service Solutions (SPSS) (IBM SPSS Statistics for Windows, Version 22.0, Armonk, NY). The Chi-square test was applied to assess the association between preoperative variables and the intraoperative score grading. We have also performed the receiver operating characteristic (ROC) curve analysis to validate the preoperative score in predicting the intraoperative findings. All tests were considered statically significant if the p-value was < 0.05.

## Results

In our study, a total of 105 patients were included in the study after fulfilling the inclusion and exclusion criteria. The mean age of patients was 57.6±16.4 years and also the patients in the age group of 40 years or more were 76.2%. The male patients were 58.1% and the remaining 41.9% were females. As per the American Society of Anesthesiologists (ASA) classification, only 1.0% of patients were having ASA IV or more and 21.0% of patients were having ASA III. The primary diagnosis was cholecystitis among 44.8% of patients and 2.9% of patients were diagnosed with GB perforation. The mean thickness of the GB wall was 3.4±1.6 mm, so a GB wall thickness of 3 mm or more was seen in 51.4% of patients during sonography. The mean diameter of the CBD was 4.8±2.2 mm, so the CBD with a diameter of <6 mm was seen in 21.9% of patients during sonography. Preoperative endoscopic retrograde cholangiopancreatography (ERCP) was done among 20.0% of patients. Among enrolled patients, laparoscopic cholecystectomy was done on an emergency basis among 2.9% of subjects (Table 3).

**Table 3:** Baseline characteristics of the patients ASA: American Society of Anesthesiologists; ERCP: endoscopic retrograde cholangiopancreatography

Variables	Frequency	%
Age (Mean+SD)	57.6±16.4 years	
Age group		
<40 years	25	23.8
40 years or more	80	76.2
Gender		
Male	61	58.1
Female	44	41.9
ASA classification		
I	36	34.3
II	46	43.7
III	22	21.0
IV or more	1	1.0
Primary diagnosis		
Choledocholithiasis	12	11.4
Gall Bladder perforation	3	2.9
Cholecystitis	47	44.8
Biliary colic	43	41.0
Gallbladder wall thickness (Mean+SD)	3.4±1.6 mm	
Gallbladder wall thickness		
3 mm or more	54	51.4
<3 mm	51	48.6
Common bile duct diameter (Mean+SD)	4.8±2.2 mm	
Common bile duct diameter		
>6 mm	23	21.9
6 mm or less	82	78.1
Preoperative ERCP		
Yes	21	20.0
No	84	80.0
Type of intervention		
Emergency	3	2.9
Elective	19	18.1
Delayed	83	79.0

**Figure 1:**
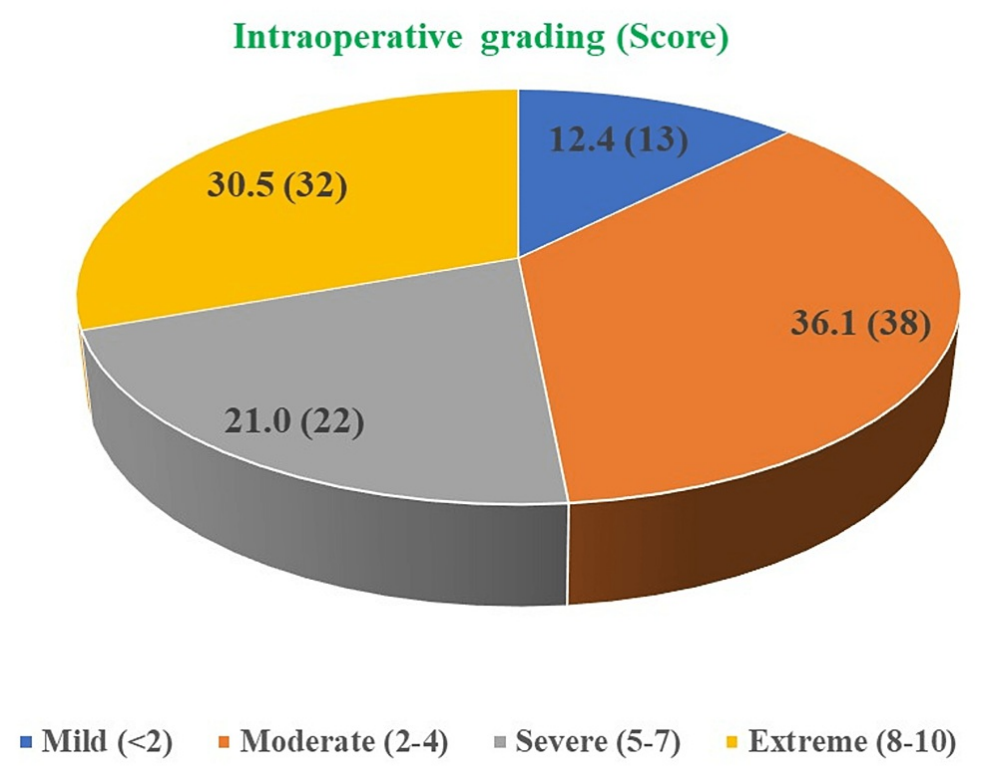
Intraoperative grading for the degree of difficulty for the procedure among patients

During the laparoscopic cholecystectomy, the degree of difficulty was assessed and it was found that among 12.4% of patients, there was a mild degree of difficulty (score <2); among 36.1% of patients, there was a moderate degree of difficulty (score 2-4); among 21.0% of patients, there was a severe degree of difficulty (score 5-7); and among 30.5% of patients, there was an extreme degree of difficulty (score 8-10) (Figure 1).

**Figure 2:**
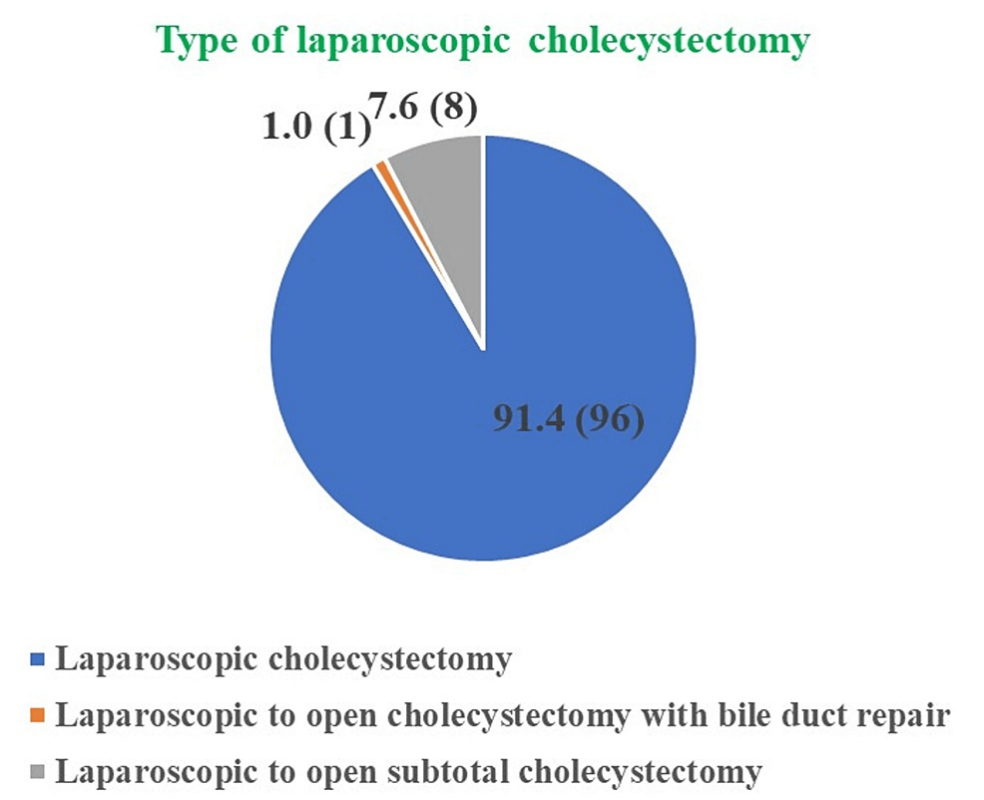
Outcome of the laparoscopic cholecystectomy performed among patients

In our study, even though the degree of difficulty among the majority of patients was severe and extreme we performed the laparoscopic cholecystectomy among 91.4% of patients and among only 1.0% and 7.6% of patients, laparoscopic to open cholecystectomy with bile duct repair and laparoscopic to open subtotal cholecystectomy was performed (Figure 2).

In our study, the groups of a mild and moderate degrees of difficulty were graded as “easy” (n=51) and groups of severe and extreme were graded as “difficult” (n=54) for the analysis. The chi-square analysis showed that the degree of difficulty as “difficult” for laparoscopic cholecystectomy was significantly (p<0.05) higher among females (57.4%) as compared to males (42.6%). Although, the degree of difficulty as “difficult” for laparoscopic cholecystectomy was higher among patients of age 40 years or more (83.3%) as compared to patients with age <40 years (16.7%) but it was statistically non-significant (p>0.05). The variables which were statistically significant with the degree of difficulty were primary diagnosis, type of intervention, ASA classification, GB wall thickness, and CBD diameter (p<0.05) (Table 4).

**Table 4:** Comparison of baseline characteristics with the degree of difficulty for the procedure among patients ASA: American Society of Anesthesiologists; ERCP: endoscopic retrograde cholangiopancreatography

Variables	Degree of difficulty frequency (%)		P value
	Difficult (n=54)	Easy (n=51)	
Gender			
Male (n=61)	23 (42.6)	38 (74.5)	0.0009
Female (n=44)	31 (57.4)	13 (25.5)	
Age group			
<40 years (n=25)	9 (16.7)	16 (31.4)	0.0771
40 years or more (n=80)	45 (83.3)	35 (68.6)	
Type of intervention			
Emergency (n=3)	3 (5.6)	0 (0.0)	0.0042
Elective (n=19)	4 (7.4)	15 (29.4)	
Delayed (n=83)	47 (87.0)	36 (70.6)	
Primary diagnosis			
Choledocholithiasis (n=12)	5 (9.3)	7 (13.8)	<0.0001
Gall Bladder perforation (n=3)	1 (1.8)	2 (3.9)	
Cholecystitis (n=47)	38 (70.4)	9 (17.6)	
Biliary colic (n=43)	10 (18.5)	33 (64.7)	
ASA classification			
I (n=36)	14 (25.9)	22 (43.2)	0.0073
II (n=46)	21 (38.9)	25 (49.0)	
III (n=22)	18 (33.3)	4 (7.8)	
IV or more (n=1)	1 (1.9)	0 (0.0)	
Preoperative ERCP			
Yes (n=21)	11 (20.4)	10 (19.6)	0.9224
No (n=84)	43 (79.6)	41 (80.4)	
Gallbladder wall thickness			
3 mm or more (n=54)	41 (75.9)	13 (25.5)	<0.0001
<3 mm (n=51)	13 (24.1)	38 (74.5)	
Common bile duct diameter			
>6 mm (n=23)	16 (29.6)	7 (13.7)	0.0485
6 mm or less (n=82)	38 (70.4)	44 (86.3)	

**Figure 3:**
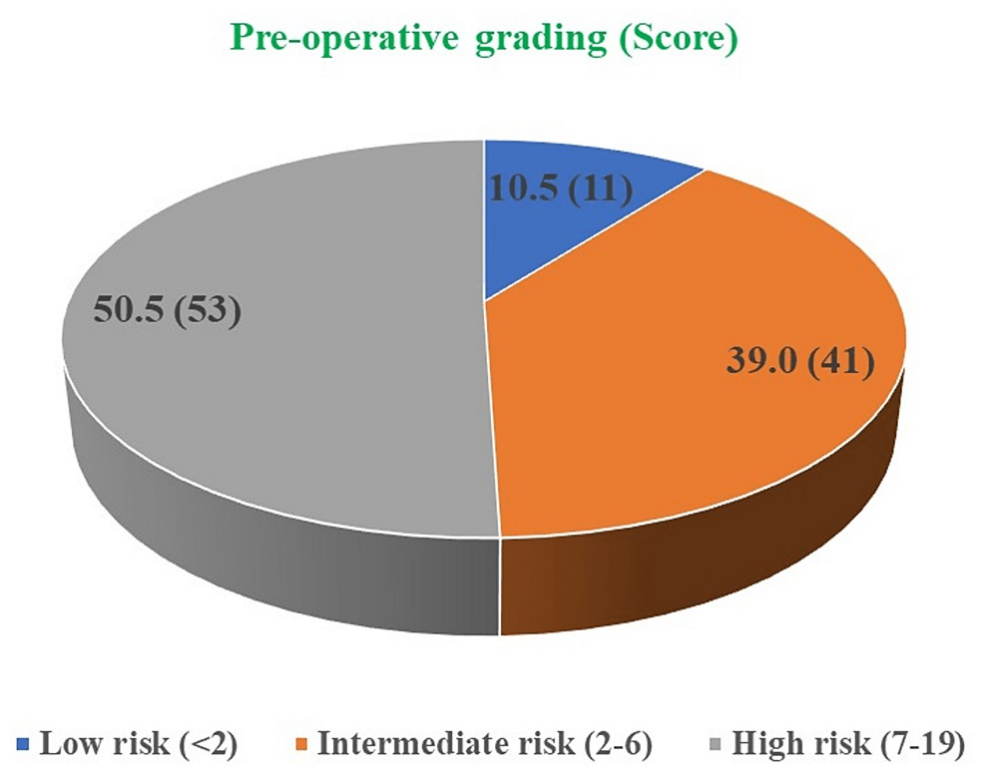
Preoperative grading for the degree of difficulty for the procedure among patients

In our study, the preoperative score was also calculated, and based on that preoperative score 10.5% of patients were categorized as “low risk” (score <2), while 39.0% of patients were graded as “intermediate risk” (score 2-6), and 50.5% of patients were graded as “high risk” (score 7-19) (Figure 3).

**Figure 4:**
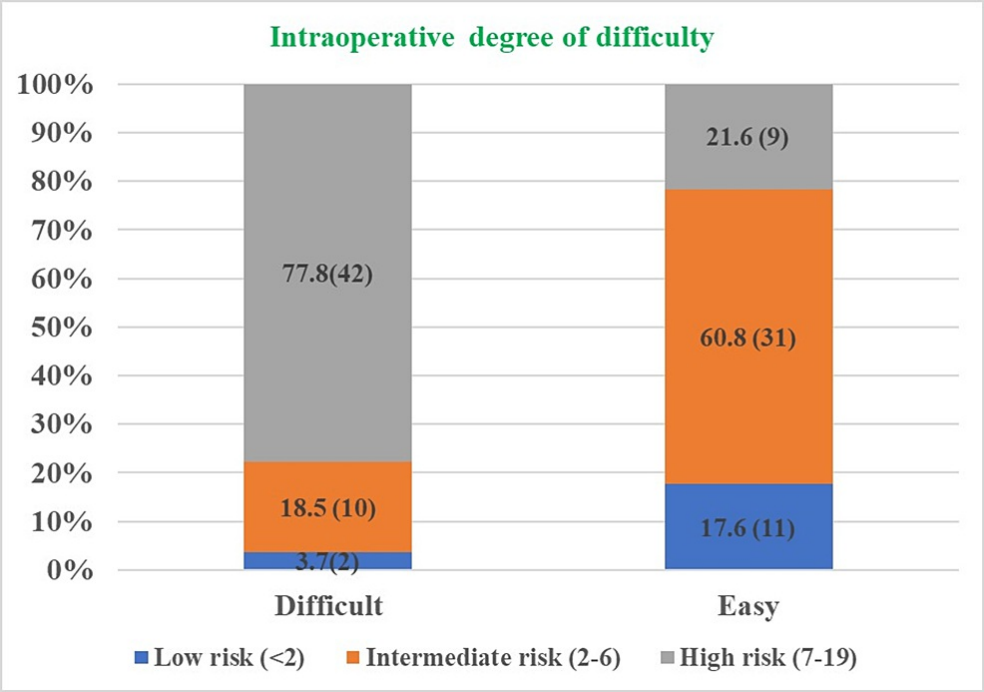
Comparison between the preoperative and intraoperative grading for the procedure among patients

We further analyzed the preoperative grading among patients based on the intraoperative grading for the degree of difficulty and it was found that 77.8% of patients (42/54) who were categorized as “difficult” were having “high risk” during preoperative grading, while 18.5% of patients (10/54) who were categorized as “difficult” were having “intermediate risk” during preoperative grading and 3.7% of patients (2/54) who were categorized as “difficult” were having “low risk” during preoperative grading (Figure 4).

**Figure 5:**
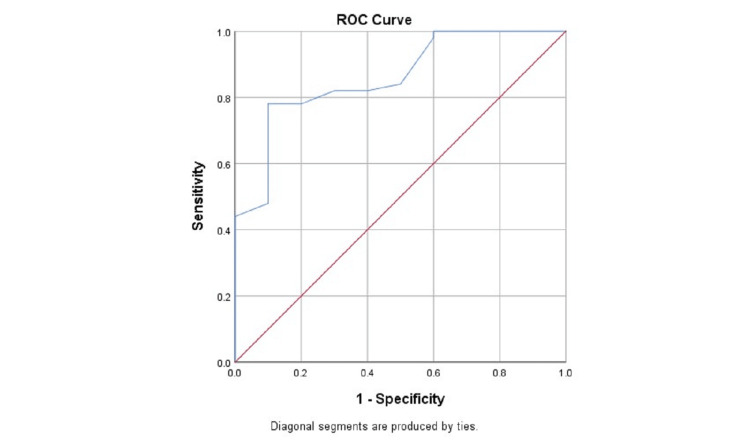
ROC for determining the validity of the preoperative score with the degree of difficulty for laparoscopic cholecystectomy among patients ROC: receiver operating characteristic

We have also analyzed the validity of the preoperative score for determining the degree of difficulty (difficult and easy) for laparoscopic cholecystectomy among patients using ROC analysis and the area under the curve (AUC) for ROC was found to be 0.860 with 95% confidence interval (CI) of 0.745-0.975 and the preoperative score was significantly valid (p<0.05) in determining the degree of difficulty laparoscopic cholecystectomy among patients (Figure 5). At the preoperative score of 6, the sensitivity and specificity for predicting easy cases were 88.2% and 73.8%, respectively and the such prediction was true in 88.6% of easy cases and 68.5.% of difficult cases.

## Discussion

As surgical technology is advancing, there is increased demand and pressure from patients and attenders to attempt laparoscopic cholecystectomy in most cases [11]. In our study, among only 1.0% and 7.6% of patients, we performed laparoscopic to open cholecystectomy with bile duct repair and laparoscopic to open subtotal cholecystectomy, so the conversion rate was 8.6% in our study. While comparing with the other studies the conversion rate of laparoscopic to open cholecystectomy ranged between 7% and 35% [12]. In our study, adhesion of dense nature from severe cholecystitis or an inability to distinguish anatomy was the major factor for the conversion of laparoscopic to open cholecystectomy, and similar causes for the conversion of laparoscopic to open cholecystectomy were observed in other studies [13,14].

In our study, we used an intraoperative scoring or grading system for the degree of difficulty during laparoscopic cholecystectomy, presented by Sugrue et al. [7], but there are also other intraoperative scoring or grading system for the degree of difficulty during laparoscopic cholecystectomy as presented by Vivek et al. [15], where some of the operative predictors were similar to the present study.

In our study, the intraoperative scoring or grading system for the degree of difficulty during laparoscopic cholecystectomy used had one operative predictor “adhesion from previous surgery limiting access” and this predictor has been shown by the other studies as an important cause for the increasing difficulty during laparoscopic cholecystectomy [4,16-20].

In our we found, that at the preoperative score of 6, the sensitivity and specificity for predicting easy cases were 88.2% and 73.8%, respectively and the such prediction was true in 88.6% of easy cases and 68.5% of difficult cases. The study by Gupta et al. showed at the preoperative score of 5, the sensitivity and specificity for predicting easy cases were 95.47% and 73.68%, respectively and the such prediction was true in 90.00% of easy cases and 88.00% of difficult cases [21].

In our study, we found that the incidence of the GB wall thickness of 3 mm or more was significantly (p<0.05) higher among patients with a degree of difficulty as “difficult” for laparoscopic cholecystectomy. Similarly, Gupta et al. and Randhawa et al. also find a significant relation between thickened GB wall (palpable GB) and intraoperative difficulty [21-23].

In our study, the variables which were statistically significant with intraoperative difficulty were gender, primary diagnosis, type of intervention, ASA classification, and CBD diameter (p<0.05) and similar variables were shown in other studies as statistically significant with intraoperative difficulty [18,24-26].

In our study, although, the degree of difficulty as “difficult” for laparoscopic cholecystectomy was higher among patients of age 40 years or more (83.3%) as compared to patients with age <40 years (16.7%) but it was statistically non-significant (p>0.05) and our findings were supported by the other studies [18,19].

Literature has shown that the conversion rate to open surgery ranges between 1% and 13% and, in our study the conversion rate was 8.6%, which is quite comparable to the available literature [27]. In our study, the conversion to open cholecystectomy was done in a total of nine cases, out of which 88.9% of cases were in the extreme intraoperative grade. None of the patients in the mild and moderate intraoperative grade were converted to open cholecystectomy (Table 5). So, the conversion to open cholecystectomy was significantly higher in difficult cases as per intraoperative grade as compared with easy cases (p<0.05). The diagnostic analysis of the intraoperative grading scale (easy vs difficult) in detecting the conversion to open cholecystectomy showed a sensitivity of 100.00% (95% CI: 66.37% to 100.00%), with a specificity of 53.12% (95% CI: 42.66% to 63.39%), and an accuracy of 57.16% (95% CI: 47.13% to 66.77%).

**Table 5:** Comparison of intraoperative grading with the conversion to open cholecystectomy

Variables	Conversion to open (%)		P value
	Yes (n=9)	No (n=96)	
Mild (<2) (n=13)	0 (0.0)	13 (13.5)	0.001
Moderate (2-4) (n=38)	0 (0.0)	38 (39.6)	
Severe (5-7) (n=22)	1 (11.4)	21 (21.9)	
Extreme (8-10) (n=32)	8 (88.9)	24 (25.0)	

Limitations

The smaller sample (n=105) can be considered as the limitation of our study. So, the studies of multicentric in nature and larger sample size are necessary to validate the present scoring system in predicting the difficult cases of laparoscopic cholecystectomy. Our unit surgeons graded the intraoperative difficulty according to findings, but the majority of cases were rated by the principal investigator, which we believe could have resulted in biasness if assessments were made by more surgeons. In our study biasness might be there as interobserver variability was not assessed.

## Conclusions

When grading the difficulties of doing a laparoscopic cholecystectomy and determining the severity of cholecystitis, this intraoperative scoring system is effective and accurate. Additionally, it signifies the need for conversion from laparoscopic to open cholecystectomy in cases of severe cholecystitis. With its use, the postoperative course could be predicted and appropriate counseling concerning the outcomes could be provided. Additionally, in our study, the preoperative scoring system was evaluated as effective and consistent in determining the difficult laparoscopic cholecystectomy.
